# “New” inhalant plant allergens*

**DOI:** 10.5414/ALX02066E

**Published:** 2020-04-23

**Authors:** Stefani T.M. Röseler, Jens M. Baron, Conny Höflich, Hans F. Merk, Murat Bas, Henning Bier†, Wolfgang Dott, Katharina Fietkau, Zuzanna Hajdu, Lorraine Kaiser, Thomas Kraus, Gottfried Laven, Silke Moll-Slodowy, Hans-Guido Mücke, Wolfgang Straff, Gerda Wurpts, Amir S. Yazdi, Adam Chaker, Galina Balakirski

**Affiliations:** 1 Department of Pneumology, Allergology, Sleep and Respiratory Medicine, Augustinians Hospital, Cologne,; 2 Department of Dermatology and Allergology, University Hospital of RWTH Aachen, Aachen,; 3 Federal Environment Agency, Section II 1.5 Environmental Medicine and Health Effects Assessment, Berlin,; 4 Otorhinolaryngology Practice, Ottobrunn,; 5 Department of Environmental Medicine, University Hospital of RWTH Aachen, Aachen,; 6 Department of Otorhinolaryngology, Helios-Amper Clinic Dachau, Dachau,; 7 Department of Otorhinolaryngology, Hospital Rechts der Isar, Technical University Munich, Munich, and; 8 Department of Dermatology and Allergology, University Hospital of Bonn, Bonn, Germany

**Keywords:** wind pollination, climate change, measuring pollen count, cypress, olive, pine, Bermuda grass, nettle, common ragweed, new inhalant plant allergens

## Abstract

Specific IgE measurements obtained from patients suffering from respiratory allergy (n = 952) show that, despite similar climatic conditions, there are clear regional differences in pollen sensitization between North Rhine-Westphalia and Bavaria. The data on sensitization levels and pollen concentration was taken from the research and development project Ufoplan 3710 61 228 of the German Environment Agency for North Rhine-Westphalia and Bavaria (2011 – 2014). Most poly-sensitized patients have already shown sensitization, both in the form of cross-reactivity and species-specific sensitization, to “new” pollen allergens, such as Bermuda grass and olive tree. These plants are currently not common in Germany, but may become considerably more widespread due to the increase in average yearly temperatures caused by the global warming. The other “new” aeroallergens discussed here are plants that can be found throughout Germany, such as nettle, cypress, and pine. Their current sensitization levels are higher than 8%; however, their clinical impact appears to be underestimated. For clinical practice it is important to identify when patients’ symptoms are typically severe and which regional plants might be responsible for the patients’ complaints in this period of time, as this affects further diagnostic strategy. Allergens having an immune effect can then be targeted by specific immunotherapies. The information on complaints of the patients should be regularly recorded in symptom diaries. Recording this information for at least 1 year may allow to discover a correlation between specific types of pollen and allergy symptoms.

*This article is dedicated to Prof. Dr. med. Henning Bier, whose humane approach to the patient and guiding principle “knowledge creates healing” had a profound and lasting impact on me. 

**German version published in Allergologie, Vol. 42, No. 2/2019, pp. 60-70**

## Introduction 

Aeroallergens mostly originate from a group of flowering plants known as angiosperms. Angiosperms evolved fairly recently, ~ 140 million years ago, in the Cretaceous period. The female ovule of the flowering plant does not leave the mother plant and is firmly connected to it, while the male gametophytes are released by the plant into the environment in the form of pollen and can, therefore, be inhaled. 

When they evolved, flowering plants were mainly dependent on animal pollination. In many cases, however, they later developed wind pollination (“secondary” wind pollination). 

“Secondary” wind-pollinated flowering plants, such as birch tree or sweet grass, make up the largest group of allergologically relevant plants. Both the “primary” and the “secondary” wind-pollinated flowering plants, unlike animal-pollinated and self-pollinating plants, share the ability to release large amounts of pollen into the air. These plants can be easily recognized by the naked eye because of their small inconspicuous flowers. Pine plants (“primary” wind-pollinated), for example, can produce ~ 1 million pollen grains per ovule (referred to as the P/O ratio) [1]. 

The pollen grain, usually referred to as “pollen” for simplicity, is a gametophyte, the carrier of the male genome. From an allergological point of view, the proteins in the pollen grain can be classified as major and minor allergens. A protein is considered a major allergen if more than 50% of sensitized patients have specific IgE antibodies to it. Major allergens, such as Bet v 1 (a major allergen of the European white birch, *Betula verrucosa*, a PR-10 protein), are phylogenetically much older than angiosperms themselves. 

Pollen concentration and plant distribution are not the only key factors in the development of allergic sensitization and allergies. The other important items to consider are the cross-reactivity between plant allergens and the clinical relevance of each plant [2, 12]. 

The pollen allergens defined as “new” in this article are:

- Pollen allergens that are not widespread in North Rhine-Westphalia (NRW) at the moment, e.g. ragweed, dog’s tooth grass (Bermuda grass), or olive tree, but which are already known as relevant allergens in their countries of origin and may be occasionally encountered in Germany. However, these plants may become progressively more common due to the increase in average yearly temperatures caused by the global warming [6, 7, 8, 19]. At the present time, patients may develop allergic symptoms without any previous contact with the plant due to cross-sensitization between, for example, mugwort and ragweed, various grasses and Bermuda grass, or ash tree and olive tree.
- Pollen allergens, such as stinging nettle, cypress, and pine plants, the allergological relevance of which was assumed to be low until now; but their current regional sensitization rates are ≥ 8%, so their clinical relevance may be underestimated.

## Patients and methods 

Parts of the data on allergic sensitization and the concentration of pollen grains/m^3^ of air were obtained from the research and development project of the German Environment Agency (Ufoplan 3710 61 228) in NRW for the years 2011 – 2014 [3]. 

People who suffered from subjective symptoms of allergic rhinitis (n = 476, age 20 – 65 years) and were exposed to local pollen (born in Germany and residing in NRW for at least 20 years at the time of the study) were included in the investigation. The personal data was collected using a modified patient questionnaire and a physician questionnaire of the DEGS study (a study on adult health in Germany) of the Robert Koch Institute (RKI). Allergological workup was performed using the GA2LEN skin prick test, spirometry, and a nasal lavage. Specific IgE diagnostic testing was performed using a microchip (ImmunoCAP^®^ ISAC V2). ImmunoCAP^®^ ISAC V2 enables serological detection of specific IgE antibodies to what is called “species-specific allergen components”, e.g., Bet v 1. The term “species-specific allergen component” is not clearly defined. Currently it is described as a substance that is related to its source in a more or less unique way and can only be found in a limited number of closely related species. Each allergen source (e.g., birch or grass pollen) may contain one or more species-specific allergen components (e.g., Bet v 1 for birch and Phl p 1 and Phl p 5 for timothy grass). 

In addition, a random sample was obtained from the above-mentioned sera (n = 476) using ImmunoCAP^®^ technology. The patients (n = 48) whose samples were analyzed showed clinical symptoms from July to September. Here sIgE levels for whole extracts of plant allergens were analyzed. The results on stinging nettle (w20) and pine (t213) are presented below. 

In the above-mentioned project, the pollen data was acquired from the German Pollen Information Service Foundation (Stiftung Deutscher Polleninformationsdienst (PID)). The data was collected by the pollen monitoring station Mönchengladbach, NRW, for the time period between 2006 – 2011, on ash, birch, mugwort, and ragweed pollen. The pollen data after 2011 was recorded by the pollen monitoring station of the University Hospital of the RWTH Aachen (pollen monitoring station Aachen-Hörn), NRW. Both of the above-mentioned stations use the monitoring method based on the Hirst principle (brand name Burkard), in accordance with the standardization guideline of the Association of German Engineers (Verein Deutscher Ingenieure, VDI) VDI 4252 Part 4, CEN prEN 16868:2018 [4, 5]. This guideline ensures the uniformity of the measurements both locally and across Europe. 

Statistical analysis of the data from the Ufoplan 3710 61 228 project was performed using Excel (Excel 2007 for Windows) and SPSS (PASW Statistics 18). The analysis of categorical data was carried out depending on the characteristics of variables using χ^2^ test or Fisher’s exact test. Pairwise group comparisons of the metric scaled variables were performed using Mann-Whitney U-test for independent samples (patient data) and Wilcoxon-test for dependent samples (pollen data). All statistical hypothesis testing was conducted on 2-sided 5% significance levels. 

## Results 

### “New” plant aeroallergens 

**Arizona cypress (Cupressus arizonica var. arizonica), family Cupressaceae**

*Distribution*

The Arizona cypress belongs to the large cypress family with worldwide distribution. It is native to the south-western United States and Mexico, but can also be found in Germany in botanical and private gardens. *Cupressus arizonica “Fastigiata”* and *Cupressus arizonica “Glauca”* are the *Cupressaceae* most commonly sold in Germany, as they are hardy and drought-tolerant. 

The cypress family comprises 7 subfamilies and 29 genera with 142 species, which are allergologically cross-reactive. The Arizona cypress belongs to the subfamily *Cupressoideae*. This subfamily also includes the Mediterranean cypress (*Cupressus sempervirens*), which is native to Iran in the eastern part of the Mediterranean region, the juniper, which is native to Germany, and the thuja (the tree of life), which is a common hedge plant in private gardens. Other allergologically relevant pollen allergens can be found in the subfamily *Taxodiaceae* (the Japanese cedar, the bald cypress) and in the subfamily *Callitroideae* (the incense cedar). 

*Flowers and pollen*

**Figure 1.**
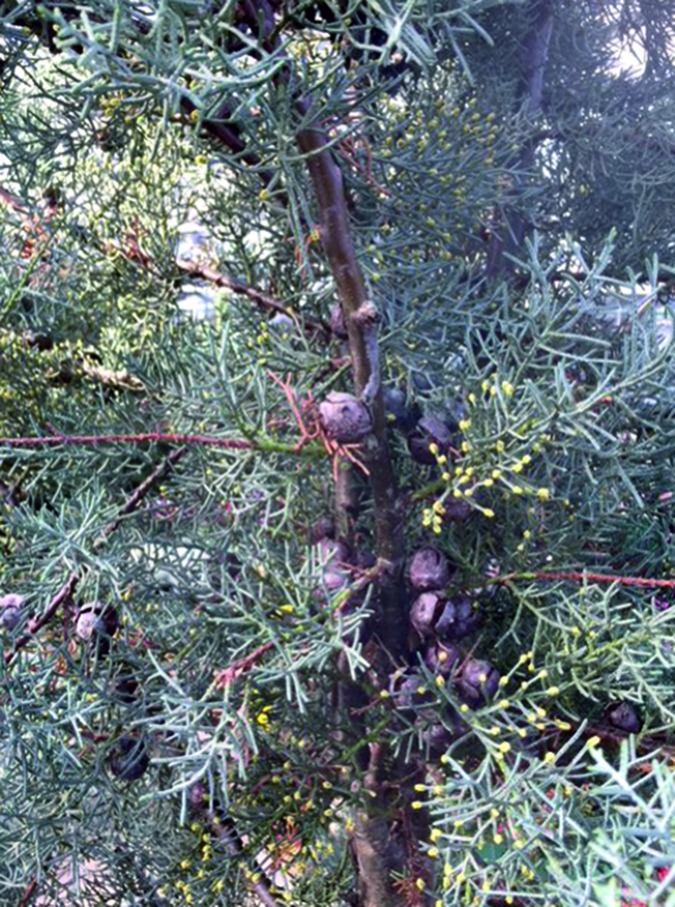
Arizona cypress (*Cupressus arizonica “Fastigiata”*) flowering, monoecious: male cones in the foreground, female cone flowers and cones with seeds in the background.

The flowering period may last from January to May, depending on the species. Pollen migration from the Mediterranean region to Western Europe has also been observed. The plant is monoecious. In the first 1 – 2 years, only the female cones develop. After 4 years, thin male cones, ~ 3 – 5 mm in size, develop at the tips (Figure 1). The pollen grains measure ~ 24 – 32 mm, are inaperturate, with star-shaped cytoplasm. 

*Currently known allergen components*

Cup a 1, Cup a 1.0101, Cup a 1.02, Cup a 2, Cup a 3, and Cup a 4. 

*Sensitization to nCup a 1 (native pectate lyase) in NRW*

Serologically, 8% (38/476) of the patients with suspected respiratory allergy had allergic sensitization to the species-specific major allergen nCup a 1. It is possible to hypothesize that this is predominantly due to the cross-reactivity to allergen components of other plants of the cypress family, as the Arizona cypress is found only rarely in Germany. In this study, allergic sensitization to cypress detected via serological investigation was found to be more frequent than to plane tree, which is an allergen recommended as part of the standardized skin prick test battery by German guidelines. 

We have additionally measured allergic sensitization rates to the species-specific allergen component nCry j 1 (the Japanese cedar), which is also a pectate lyase. From the patients with suspected inhalation allergies, 4% (19/476) showed allergic sensitization to this protein. 

*Cross-reactivity*

There is a high cross-reactivity within the cypress family. Cross-reactivity to mugwort and ragweed has also been described. 

**Olive (Olea europaea), family Oleaceae**

*Distribution*

The olive tree is the oldest and most important cultivated tree in Europe. It is grown in the Mediterranean countries, the Middle East, and South Africa. In Germany at the present time large-scale olive tree cultivation has failed. Therefore, it is grown almost exclusively in private gardens and as an ornamental plant. 

*Flowers and pollen*

**Figure 2.**
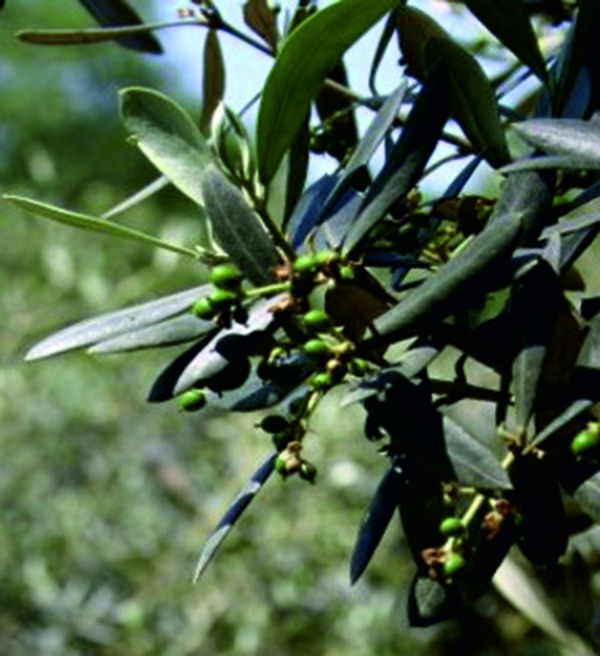
Olive, flowering and fruiting.

In the Mediterranean countries, the flowering period of the olive is from late April to June. Its predominantly hermaphrodite flowers stand in panicles, with 10 – 40 flowers at the end or side of the branch (Figure 2). The pollen grain is ~ 24 mm in size, roundish to triangular, oblate, isopolar, reticulate. In Germany, olive pollen is not monitored systematically because of its rare occurrence. Olive pollen (Ole e 1) shows cross-reactivity to ash pollen (Fra e 1) [13], which in 2006 – 2011 in Germany had a median amount of 1.033 pollen grains/m^3^ air and a median occurrence of 43 days per year. The flowering period of ash in NRW is before the birch blossom in April [20]. 

*Currently known allergen components*

Ole e 1, Ole e 2, Ole e 3, Ole e 4, Ole e 5, Ole e 6-15, and their isoforms. 

*Sensitization in NRW to nOle e 1 (trypsin inhibitor-like protein)*

19% (90/476) of the patients with suspected inhalant allergy had allergic sensitization to the native Ole e 1 (trypsin inhibitor-like protein), which was detected serologically. 17% (80/476) of the patients with suspected inhalant allergy had a positive result to olive pollen in the skin prick test. 7% (32/476) had allergic sensitization to olive without a positive result to ash in the skin prick test [3]. 0.6% of allergic patients demonstrated allergic sensitization to the allergen component rOle e 9 (recombinant 1,3-β-glucanase). This allergen component is associated with high exposure to olive pollen, bronchial asthma, and the latex-fruit syndrome (an allergic reaction to latex, avocado, kiwi, banana, and chestnuts) [13]. 

*Cross-reactivity with ash tree and grasses*

In NRW, 29% of allergic patients had a positive result to ash in the skin prick test. Of these, 19% had allergic sensitization to ash pollen only and had a negative result to olive in the skin prick test [3]. This makes it possible to speculate that in Germany insect-pollinated plants, such as lilac, forsythia, privet, and jasmine, may to a certain extent contribute to such high numbers of patients with allergic sensitization. These plants can cause higher pollen levels locally but are insect-pollinated and have sticky pollen and a lower number of pollen grains per ovary. 

**Monterey pine (Pinus radiata), family Pinaceae**

*Distribution*

Originally from California, this tree is being increasingly planted in the Mediterranean region for rapid afforestation and as a timber source. Because of its rapid growth, it is planted in forests all over the world. In Germany it is rare and can be found as an ornamental plant and in botanical gardens. However, it belongs to the Pine family, which contains ~ 120 species of coniferous trees, which are widespread in Germany and the Northern Hemisphere, both in the nature and in forestry. 

*Flowers and pollen*

The flowering period of the Monterey pine is from April to May. As mentioned above, pines are primarily wind-pollinated and, therefore, produce large amounts of pollen (~ 1 million pollen grains per ovary). The male cones grow in groups at the base of the branches. The pine’s relatively large pollen (~ 65 mm in size) has two easily recognizable air-filled sacs (the radius of the sphere is ~ 15 mm); the sacs are reticulate. 

*Currently known allergen components*

Allergen components have not been identified for *Pinus radiata* yet. 

*Sensitization rates in NRW*

In the sample of the 48 patients with suspected inhalant allergy, 12% had allergic sensitization to Monterey pine (tested with the total extract). It is possible to hypothesize that it is not a primary sensitization to *Pinus radiata*, but allergic sensitization to other pine plants or other cross-reactive species (see above). 

The high sensitization rate was surprising, especially because we investigated patients who exhibited symptoms between July and September, while pine plants bloom between April and May. In the current investigation, the authors explored only the possible cross-reactivities between nCup a 1 and nCry j 1 in the examined patient collective. To what extent these sensitizations are species-specific, clinically relevant, or cross-reactive (for example, to ryegrass (*Lolium perenne*) pollen) needs to be investigated. 

It is also important to investigate possible cross-reactivity to colophony (rosin), when it takes the form of type IV hypersensitivity reactions. Colophony, which is produced from pine resin, is a common contact allergen and can cause severe type I hypersensitivity reactions, such as bronchial asthma, when inhaled. 

*Cross-reactivity*

There is a significant cross-reactivity with other pines, but also with cypresses, olives, possibly ryegrass. 

**Dog’s tooth grass syn. Bermuda grass (Cynodon dactylon), family Poaceae**

*Distribution*

**Figure 3.**
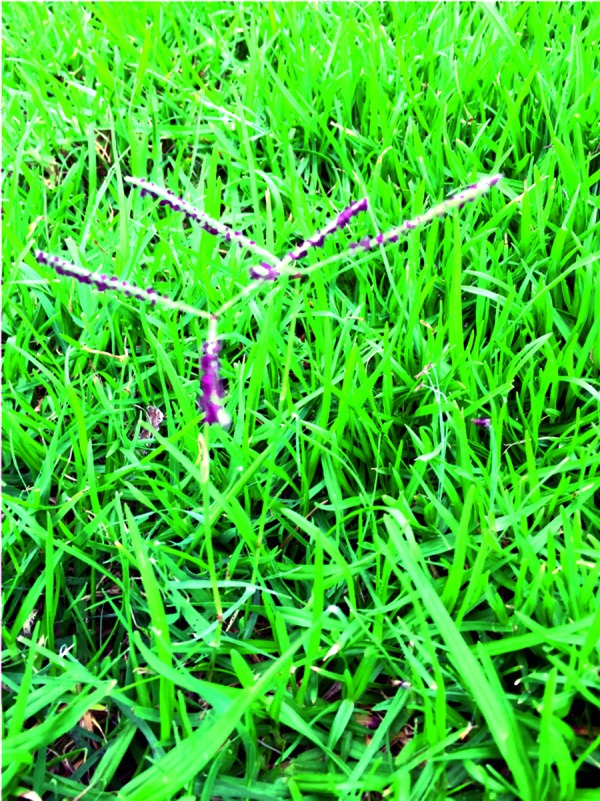
Dog’s tooth grass (Bermuda grass).

Dog’s tooth grass has successively spread from the tropics in the temperate zones. It is a creeping, drought-resistant grass that can be found in Europe, especially in the Mediterranean region. It has also been described to appear in the wine-producing areas of Germany. It can be easily recognized by the naked eye (Figure 3). Seeds of *Cynodon dactylon* can be purchased in Germany. 

*Flowers and pollen*

The flowering period of dog’s tooth grass lasts from May to September; flowers may occasionally appear until the winter months. Usually 3 – 7 ears (spikes) grow out horizontally from a central stem. The pollen grain of dog’s tooth grass is 33 μm in size, round to oval, operculate. In the period from 2014 to 2018 a median of 4,604 grass pollen/m^3^ of air per year was detected at the pollen monitoring station in Aachen-Hörn. 

*Currently known allergen components*

Cyn d 1, Cyn d 2-24. 

*Sensitization rates in NRW*

42% of the patients from NRW who suffered from airborne allergies had allergic sensitization to the native species-specific major component Cyn d 1 of dog’s tooth grass. Thus, the sensitization to dog’s tooth grass was the second most common sensitization to grass allergen components in the ImmunoCAP^®^ ISAC testing, after the locally common timothy grass component Phl p 1. It is possible that this high sensitization rate is caused by cross-reactivity between Phl p 1 and Cyn d 1. Both species-specific allergen components belong to group I grass pollen allergens. 

*Cross-reactivity*

High allergic cross-reactivity between the pollen of different grasses is well known, especially among group I grass pollen allergens. Current studies in the field of molecular allergology estimated cross-reactivity of ~ 85% between dog’s tooth grass and timothy grass [8], which corresponds to the clinical findings of our investigation. However, this also supports the argument that allergens that are scarcely or not at all present in Germany might become clinically relevant “new” allergens because of cross-reactivity. 

**Stinging nettle (Urtica dioica), family Urticaceae**

*Distribution*

The stinging nettle is a widespread perennial plant that grows on moist, nitrogen-rich loam, and clay soil, especially in neglected areas. Historically, it has always grown close to human settlements. In Germany, the burning nettle (*Urtica urens*) is also widespread. Pellitory (*Parietaria*) also belongs to the *Urticaceae* family. While it grows mainly in the Mediterranean area, smaller amounts can also be found in the Rhine valley [4]. 

*Flowers and pollen*

The male stinging nettle has upright inflorescences and the female hanging ones, which are wind pollinated. The flowering period lasts from June to September. At many pollen monitoring stations in Central Europe, nettle pollen and pellitory pollen are the most abundant herbal pollen types. The pollen (10 – 25 μm) of the pellitory cannot be differentiated microscopically from pollen of the stinging nettle. The pollen grains are round to elliptical, with small pores. 

*Currently known allergen components*

Allergen components of the stinging nettle have not been identified yet. 

**Figure 4. Figure4:**
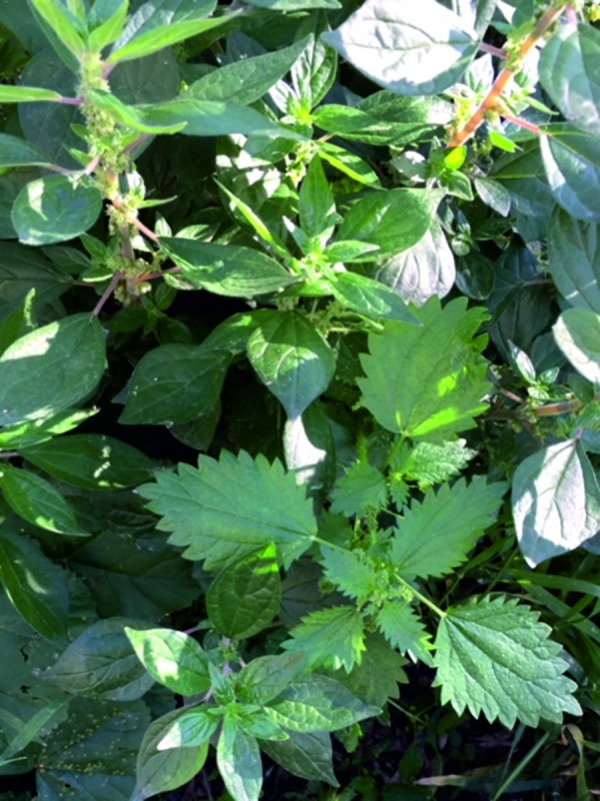
Pellitory on the left, stinging nettle on the right in the foreground. Simultaneous occurrence of different plants of the *Urticaceae* family in one location is rare.

For pellitory: Par j 1 (LTP), Par j 2 (LTP), Par j 3 (profilin), Par j 4 (calcium-binding protein). [Fig Figure4]

*Sensitization rates in NRW*

In the above-described random sample (n = 48), 23% of the patients with suspected airborne allergy and clinical symptoms from July to September showed allergic sensitization to nettle pollen. In approximately half of the patients with allergic sensitization to nettle pollen, the sIgE levels to nettle were higher than to any other pollen types that typically cause clinically significant sensitization in this time period. 

*Cross-reactivity*

Cross-reactivity within the *Urticaceae* family has been described, as well as with elm tree. None of the patients from the investigated sample with allergic sensitization to nettle had allergic sensitization to Par j 2, the species-specific allergen component of pellitory. 

**Ragweed (Ambrosia artemisiifolia), family Asteraceae**

*Distribution*

This rather invasive plant originates from North America [21], but due to the climate change it is spreading all over Europe and can be detected increasingly in Hungary, Slovenia, Slovakia, and Romania. The plants are possibly brought in by birds and are described to be common at loading docks in port facilities, railway stations, and airfields. 

*Flowers and pollen*

The female and male flowers occur separately at different parts of the same plant and are upright, in contrast to the similar-looking common mugwort. For the first time, measurable ragweed pollen concentrations were found in almost all federal states of Germany in 2008. The ragweed pollen concentration between 2006 and 2011 had a median of 5 pollen grains/m^3^ of air per year. In 2017, more than 100 ragweed pollen grains/m^3^ of air per year were measured for the first time at the pollen monitoring station Aachen-Hörn, NRW. The spherical pollen grains are ~ 20 mg in size and the pollen wall is spiked and usually has one to several pores at the equatorial axis. Even the smallest amounts of ragweed pollen can cause clinical symptoms in allergic individuals [9]. 

*Currently known allergen components*

Amb a 1 (pectate lyase), Amb a 2-12. 

*Sensitization rates in NRW*

None of the patients with suspected respiratory allergy (n = 476) showed any sensitization to the species-specific allergen component Amb a 1; however, 19% had a positive skin prick test result to the total pollen extract. 7% of the patients who tested positive to ragweed in the skin prick test had a negative skin prick test result to mugwort, which could indicate possible cross-reactivity with other pollen allergens [3]. 

*Cross-reactivity*

There is allergic cross-reactivity to the pollen of the *Asteraceae* family, for example, mugwort, chamomile, arnica, marguerite, chrysanthemum, goldenrod, tarragon, sunflower, dandelion, chicory, endive, artichoke, Jerusalem artichoke (sunroot), lettuce, black salsify, and yacon. 

## Discussion 

### Allergic sensitization to pollen 

The data on regional allergens and sensitization rates in Germany is limited. The Federal Environment Agency, the University Hospital of the Technical University of Munich, and the University Hospital of RWTH Aachen University have published the first reports on sensitization to ash, birch, olive, mugwort, and ambrosia in patients who suffered from respiratory allergy in NRW and Bavaria. Data analysis showed unexpected regional differences in allergic sensitization which could not be explained by cross-reactivity to pollen pan-allergens, non-exposure variables, or by plant distribution and pollen load [3]. 

Here is a brief summary of the study data on regional differences and the “new” plant aeroallergens compiled from current publications [3, 10, 11]:

- Allergic sensitization to *Ambrosia* (ragweed) was not detectable serologically (end of study: 2014) using the species-specific allergen component (Amb a 1) in NRW and in Bavaria. However, comparative analysis of the data of NRW and Bavaria showed a statistically significantly higher number of patients in NRW (19%) than in Bavaria (11%) who had a positive skin prick test result to *Ambrosia artemisiifolia* (p < 0.05). This could not be explained by allergic sensitization to mugwort, whose levels were similar in the two federal states both in skin prick testing and in sIgE levels to the species-specific component Art v 1. As expected, the presence of allergic sensitization to ragweed currently implies the presence of allergic sensitization to mugwort, but not vice versa.
- Allergic sensitization to olive (Ole e 1) was statistically significantly more common in Bavaria than in NRW when investigated serologically (27% versus 19%) and via skin prick testing (37% versus 17%). The cross-reactivity between the allergen components Ole e 1 (the species-specific component of olive) and Fra a 1 (the species-specific component of ash) is reported to be 88% [14]. In Bavaria, 84% of the patients with allergic sensitization to ash also showed allergic sensitization to olive pollen. However, in NRW only 35% of the patients with allergic sensitization to ash pollen had allergic sensitization to olive pollen as well. In NRW, therefore, these sensitization levels must have been caused by other allergen components of ash and/or other plant pollen.

In addition to the above mentioned pollen allergens with well-known species-specific allergen components, a large number of local pollen allergens can only be used for diagnostic purposes in the form of total extracts or even actual plant tissues, if no standardized commercial extracts are available. These extracts, however, contain a mixture of a large number of species-specific allergens and nonspecific ones, also referred to as pan-allergens. 

In the group of allergic patients whose symptoms primarily manifested from July to September (n = 48) the initial sensitization levels were measured via total pollen extracts testing (stinging nettle and pine). However, further testing revealed that 10% of those sensitized to nettle also had allergic sensitization to pan-allergens CCD, profilin, and polcalcin. The same was true in ~ 4% of those sensitized to pine pollen. Thus, the species-specific and/or cross-reactive sensitization to the pollen allergens investigated here, calculated without concomitant allergic sensitization to pan-allergens, was 12% for stinging nettle and 8% for pine pollen in the time frame in which this group of patients reported clinical symptoms. 

### Pollen load 

According to unpublished data (M. Werchan, K.C. Bergmann, The German Pollen Information Service Foundation, 2017), more yew tree pollen and pollen of the cypress family was collected at southern pollen monitoring stations and more nettle pollen at the northern ones in the years 2015 and 2016. 

In contrast to the German Pollen Information Service Foundation data, the information discussed above in the “Flowers and Pollen” sections was collected by isolated pollen monitoring stations and thus is only partially representative for the entire regions of Bavaria and NRW. These pollen monitoring stations register the pollen load within a certain radius around the station, depending on the type of pollen and the weather conditions. In order to collect more accurate local pollen data, a network of several pollen monitoring stations is required so that positional factors, for example, a chestnut tree park located near the measuring point, would not affect the calculations and make real regional differences apparent [4, 5, 15]. As there is a lack of pollen monitoring stations in many regions, producing a nationwide report on pollen load in Germany is not currently possible. The reason behind this is that, unlike bacteria, viruses, and fungi, allergens are perceived as not harmful or pathogenic. This leads to a situation in which allergological research facilities are underfunded and understaffed, and pollen monitoring stations are operated almost exclusively by volunteer researchers. The stations are located in those areas in which the volunteers can maintain them; not in the ones that have large quantities of allergens or unusual sensitization patterns. The pollen monitoring stations form the pollen monitoring network in Germany in cooperation and with support of the German Pollen Information Service Foundation. 

Based on the findings discussed above, it appears highly plausible that sensitization to these supposedly “new” aeroallergens has already affected, more or less severely, a large proportion of the 11 million German citizens who suffer from pollen allergies [16, 17, 18]. 

In addition, the presence of local pollen allergens may cause clinical allergological symptoms to “new allergens” due to the cross-reactivity. This article provided examples of such an effect with the following “new” allergens: olive, ragweed, and dog’s tooth grass (Bermuda grass). Therefore, these allergens can become clinically relevant before becoming established as a part of the local plant landscape. 

Clinically, all of the above means that it is important to collect specific patient history if allergic rhinitis is suspected. The physician should not trust the patient’s self-diagnosis (“I suffer from birch/grass allergy”), especially in polysensitized patients with multiple allergies. Although it is difficult for many patients to be precise, the physician should specifically inquire about the period during which complaints persist and about the most severe intervals. When the period of symptom manifestation is known, a set of available diagnostic tools can be used, with critical interpretation of the results. Here, accurate data on local pollen (if available) is crucial for formulating a diagnostic strategy. Additionally, if the diagnosis is made with the use of total pollen extracts, possible sensitization to pan-allergens, such as profiline or polcalcine, should always be considered. 

### Regional pollen measurements in Germany available online 

Pollen count in Northern Germany: http://www.pollenflug-nord.de/ (retrieved: March 10, 2020) 

Pollen count in Aachen: https://www.ukaachen.de/kliniken-institute/aachener-comprehensive-allergy-center-acac/polleninfos/pollenmessung-aachen.html (retrieved: March 10, 2020) 

Pollen count in Cologne under development: https://www.severinskloesterchen.de/medizin/pneumologie-allergologie-schlaf-und-beatmungsmedizin/leistungsspektrum/allergologie (retrieved: March 10, 2020) 

Electronic Pollen Information Network of Bavaria (ePIN), currently under construction: https://epin.lgl.bayern.de/pollenflug-aktuell (retrieved: March 10, 2020) 

### Pollen forecasts for Germany available online 

The German Pollen Information Service Foundation (Stiftung Deutscher Polleninformationsdienst (PID)): http://www.pollenstiftung.de/pollenvorhersage/ (retrieved: March 10, 2020) 

The German Weather Service (Deutscher Wetterdienst (DWD)) (Pollen data from the German Pollen Information Service Foundation and DWD pollen monitoring station Freiburg): https://www.dwd.de/DE/leistungen/gefahrenindizespollen/gefahrenindexpollen.html (retrieved: March 10, 2020) 

## Funding 

A part of the study was funded by the Germany Federal Ministry for the Environment, Nature Conservation, Building and Nuclear Safety (Ufoplan research grant number FKZ 3710 61 228).****

## Conflict of interest 

Dr. S. Röseler received research funding as well as speaking and counseling fees from Alk-Abelló Arzneimittel GmbH, Allergopharma GmbH & Co. KG, Bencard Allergie GmbH, HAL Allergie GmbH, LETI S.L., Novartis Pharma GmbH, and ROXALL Medizin GmbH. 

Dr. G. Balakirski received travel grants from Alk-Abelló Arzneimittel GmbH and Novartis Pharma GmbH as well as speaking fees from Novartis Pharma GmbH.
